# Rethinking Clinical Trials of Transcranial Direct Current Stimulation: Participant and Assessor Blinding Is Inadequate at Intensities of 2mA

**DOI:** 10.1371/journal.pone.0047514

**Published:** 2012-10-17

**Authors:** Neil E. O’Connell, John Cossar, Louise Marston, Benedict M. Wand, David Bunce, G. Lorimer Moseley, Lorraine H. De Souza

**Affiliations:** 1 Centre for Research in Rehabilitation, School of Health Sciences and Social Care, Brunel University, London, United Kingdom; 2 Research Department of Primary Care & Population Health, University College London, London, United Kingdom; 3 School of Physiotherapy, University of Notre Dame Australia, Fremantle, Australia; 4 Centre for Cognition and Neuroimaging, Brunel University, London, United Kingdom; 5 Sansom Institute for Health Research, University of South Australia, Adelaide, & Neuroscience Research Australia, Sydney, Australia; The James Cook University Hospital, United Kingdom

## Abstract

**Background:**

Many double-blind clinical trials of transcranial direct current stimulation (tDCS) use stimulus intensities of 2 mA despite the fact that blinding has not been formally validated under these conditions. The aim of this study was to test the assumption that sham 2 mA tDCS achieves effective blinding.

**Methods:**

A randomised double blind crossover trial. 100 tDCS-naïve healthy volunteers were incorrectly advised that they there were taking part in a trial of tDCS on word memory. Participants attended for two separate sessions. In each session, they completed a word memory task, then received active or sham tDCS (order randomised) at 2 mA stimulation intensity for 20 minutes and then repeated the word memory task. They then judged whether they believed they had received active stimulation and rated their confidence in that judgement. The blinded assessor noted when red marks were observed at the electrode sites post-stimulation.

**Results:**

tDCS at 2 mA was not effectively blinded. That is, participants correctly judged the stimulation condition greater than would be expected to by chance at both the first session (kappa level of agreement (κ) 0.28, 95% confidence interval (CI) 0.09 to 0.47 p = 0.005) and the second session (κ = 0.77, 95%CI 0.64 to 0.90), p = <0.001) indicating inadequate participant blinding. Redness at the reference electrode site was noticeable following active stimulation more than sham stimulation (session one, κ = 0.512, 95%CI 0.363 to 0.66, p<0.001; session two, κ = 0.677, 95%CI 0.534 to 0.82) indicating inadequate assessor blinding.

**Conclusions:**

Our results suggest that blinding in studies using tDCS at intensities of 2 mA is inadequate. Positive results from such studies should be interpreted with caution.

## Introduction

Transcranial direct current stimulation (tDCS) is a non-invasive method of electrical stimulation of the cortex and has been shown to modulate brain activity specific to the site and parameters of stimulation [1]. tDCS research has ranged from the investigation of its physiological effects on brain activity and function [1], to a range of clinical applications, most notably chronic pain [2]–[5], depression [6] and stroke [7].

Clinical evaluation of tDCS is considered superior to that of other non-invasive brain stimulation methods because the stimulation condition can be concealed through the use of a sham condition [8]. Sham tDCS involves an identical process to active stimulation but, without the participant’s knowledge, the stimulator is switched off after around 30 seconds. Gandiga, et al. [9] reviewed the results of two crossover studies involving participants undergoing tDCS at 1 mA intensity or sham and found that the stimulation condition could not be distinguished by participants or blinded investigators. Ambrus et al. [10] highlighted the benefit of naivety by showing that, although even experienced participants could be effectively blinded using 1 mA or sham, when the participant was an experienced tDCS investigator, blinding was less effective. That is, even with low intensity tDCS, naivety is important for effective blinding from sham.

Many clinical trials involving tDCS use 2 mA stimulation and presume effective blinding [for example 2,3]. Largely on the basis of these trials, tDCS is considered a very promising therapeutic tool. tDCS at 2 mA is associated with more sensory effects than tDCS at 1 mA [11], which clearly casts doubt over assumption of effective blinding. Almost all trials of tDCS for chronic pain identified in a recent Cochrane review [12] used stimulation intensities of 2 mA. Only one parallel study [5] reported difficulties with participant blinding and even then did not elaborate on those difficulties. Conversely two parallel trials of tDCS for depression reported that effective treatment masking was maintained [13], [14]. Our group recently undertook a clinical crossover study [15] of tDCS at 2 mA for chronic back pain and found that participants may have been able to distinguish between the active stimulation and sham condition and a crossover study of tDCS for food cravings has similarly reported inadequate blinding [16].The use of a crossover design might be particularly problematic because exposure of participants to both active stimulation and sham increases the likelihood that they will distinguish one as more credible than the other. In addition to concerns over participant blinding, blinded assessors may also be able to distinguish between conditions because of skin redness that is observable at the electrode sites, primarily under the reference electrode, after active but not sham stimulation [11]. Given the growing endorsement of 2 mA tDCS as a therapeutic tool, it is remarkable that the assumption that such studies are effectively blinded has not been formally tested. The need for such testing was recently highlighted in a review of challenges for tDCS research [11]. We aimed to determine how well 2 mA tDCS can be blinded against a sham stimulation condition. Our primary research questions were “At stimulation intensities of 2 mA, do people correctly judge the true stimulation condition more than would be expected by chance and is this judgement influenced by previous exposure to sham or real stimulation?” and “At stimulation intensities of 2 mA, is skin redness at the visible electrode site more visible to the blinded assessor following active stimulation than it is following sham?”.

## Methods

This study had full approval from the School of Health Sciences and Social Care Research Ethics Committee, Brunel University and conformed to the Helsinki declaration. All participants gave written informed consent. This study used a double-blind randomised crossover design.

### Participants

Healthy individuals aged over 18, were recruited from staff and students at Brunel University, and their family and friends. Participants must have had no prior experience of tDCS stimulation. Exclusion criteria were prior/existing history of neurological disease, psychiatric disorder, dyslexia, diabetes, epilepsy, head injury, musculoskeletal or neurological injury to the limb, dermatological condition affecting the scalp, poor understanding of written English or any other communication impairment.

### Recruitment

To establish conditions that best reflected what might occur in clinical trials of tDCS, participants were misdirected regarding the true research question. We misinformed participants that the study aimed to investigate the potential effects of tDCS on a word memory recognition task. The purpose of this deception was to reduce the likelihood that participants would afford more attention to distinguishing between the active stimulation and sham conditions than they might during a clinical trial of tDCS. However in the event that blinding was found to be adequate we planned to perform a formal analysis of the effect of tDCS on performance of this task. Although participants were informed that the study would involve both real and sham conditions, they were not informed about the true research question. We made this deception clear in our application for ethical approval and received approval to proceed in this way.

### Outcomes

The primary outcomes were:

1. The participant’s YES/NO answer to the question “Do you feel that you have just received the real brain stimulation?”
2. A 10 cm Visual Analogue Scale (VAS) of the participant’s confidence in that judgement, worded as follows “Please place a mark on the line below that best represents your level of confidence in that judgement.” The left anchor was labelled “not confident at all” and the right anchor was labelled “completely confident”.

To answer the second research question the assessor documented every occasion the participant had noticeable skin redness at the visible electrode site(s) following stimulation. This was documented as a simple YES/NO response. No formal threshold of skin redness was used as we wished to simply note when the assessor might be aware of noticeable redness in a clinical trial.

We did not question participants further regarding their perceptions or sensations during or after the stimulation to avoid making this the focus of their attention during the study.

### Procedure

All eligible participants were randomised to an order of stimulation (active followed by sham, or vice versa). The randomisation schedule for all participants was established prior to recruitment by an independent administrator using a computer generated random numbers sequence (http://www.randomizer.org/). A randomly generated list of numbers 1 and 2 was generated (1 = active stimulation first, 2 = sham stimulation first) and each of these numbers was sealed in an opaque envelope with a corresponding participant number. The corresponding envelope was accessed for each consecutive participant on the day of the first stimulation session by the sole unblinded researcher who delivered the stimulation (JC) and who had no involvement in the recruitment or assessment process. Neither the participant nor the assessor (NO’C) were informed of the stimulation order and the randomisation code was maintained until all participants had completed the study.

Participants visited the laboratory twice with a minimum 2 week washout period between visits. At each visit, participants completed the word memory task, and then received their stimulation (active or sham). Participants were then asked to report any adverse events and this was followed by a repeat of the word memory task. Participants then completed the form concerning their judgement of the stimulation condition. Although it was the primary research question, this form was undertaken at the end of the visit to appear secondary. The question, and the participant’s response, was discussed in no more detail than that required for successful completion.

### tDCS Stimulation

tDCS was delivered using a battery driven CX-6650 ramp controlled DC stimulator (Rolf Schneider Electronics, Germany). Current was delivered by electrodes encased in sponge pads (35 cm^2^) soaked with 0.1% (140 mMol) saline solution. The machine was kept behind the participant and was out of the view of both the participant and the blind assessor for the entire stimulation period. For both the active and sham conditions, the anode was placed over the left motor cortex of the subject and the cathode was placed over the contralateral supraorbital region. Electrodes were secured using soft elastic straps. The location of the motor cortex was estimated using the international 10–20 EEG system, with the centre of the electrode pad located 1 cm anterior and 4 cm lateral to the vertex.

In the active stimulation condition, a constant current of 2 mA intensity (current density 0.057 mA/cm2) was applied for 20 minutes, with a 5 second ramping phase at the beginning and end of stimulation. In the sham stimulation condition, the machine was activated using identical parameters but was switched off without the participant’s knowledge after 30 seconds. The researcher who applied the stimulation recorded the voltage levels 30 seconds after the onset of stimulation.

### The Memory Task

In order to maintain the impression that the study aimed to test the effects of tDCS on memory we used a standard word recognition test, which was performed by the participants on a laptop computer using E-Prime software (©Psychology Software Tools, Sharpsburg USA).

### Data Analysis

For the primary analysis, the data from each session were analysed separately to answer the research question for parallel and crossover study designs. Analyses were performed using IBM SPSS version 18 statistical software.

We used the Kappa measure of agreement (κ) to test whether participants successfully judged the stimulation condition more than would be expected by chance and to test whether the assessor noticed a visible redness following stimulation on the skin under the electrode sites more commonly after active stimulation than they did after sham. This was in almost all cases noticed under the reference electrode. Cut-offs for characterizing the level of agreement were <0.2 poor, 0.21–0.4 Fair, 0.41–0.6 Moderate, 0.61–0.8 Good, 0.81–1 very good [17].

We investigated differences in participants’ confidence about their judgements with the following factors: stimulation condition (active/sham), participant’s judgements regarding whether they had received active stimulation (yes/no) and session number (first or second) using the appropriate non-parametric test. We accepted statistical significance for all tests at α<0.05.

## Results

We recruited 100 participants (75 female). The mean (SD) age was 24 (8.3), range 18–62. Fifty-four participants were randomly allocated to receive active stimulation followed by sham. Ninety-nine participants completed the first stimulation session in full. One female participant withdrew from the study in the first session because they could not tolerate the stimulation. Three participants (2 female) did not attend for a second session: two stated that they were too busy to participate further and one did not respond to correspondence. We obtained complete data from 96 participants.

### Methodological Checks

Participants’ confidence ratings and the initial stimulation voltage were not normally distributed.

### Participant Blinding

Table 1 presents the data for participants’ judgements of the stimulation condition.

**Table 1:** Participant’s judgements of the stimulation condition, for each stimulation condition and session.

Participants Judgements		Stimulation condition		Totals
		Active Stimulation	Sham Stimulation	
**Session 1**	**Judged “yes”**	39	20	59
	**Judged “no”**	15	25	40
	**Totals**	54	45	99
**Session 2**	**Judged “yes”**	39	6	45
	**Judged “no”**	5	46	51
	**Totals**	44	52	96

(“Yes” reflects a judgement of active stimulation, “No” reflects a judgement of sham stimulation.).

#### Session one

72% of participants who received active stimulation, and 56% of participants who received the sham, correctly judged the stimulation condition. Overall, 65% of participants correctly judged the stimulation condition they received which represents a “fair” level of agreement (κ = 0.28, 95% confidence interval (CI) 0.09 to 0.47 p = 0.005).

#### Session two

89% of participants who received active stimulation and 88% of participants who received sham judged correctly, which represents a “good” level of agreement (κ = 0.77, 95%CI 0.64 to 0.90), p<0.001).

### Participant Confidence

Participants’ confidence in their judgement of the stimulation condition was significantly higher in the second stimulation session (median (IQR) 6.55 (1.85 to 7.3) than it was the first stimulation session (5.6 (3.77 to 8.48)) (Wilcoxon signed rank test, p<0.001). Confidence was higher where participants judged they received active stimulation in the first session (median (IQR) judged “Yes” 6.4 (2.3 to 7.9), judged “No” 3.050 (1.65 to 6.65), Mann Whitney U test p = 0.028) but not in the second stimulation session (judged “Yes” 7 (5.25 to 8.8), judged “No” 6 (2.7 to 8), p = 0.173).

### Assessor Blinding

Table 2 presents the frequency that the assessor noticed skin redness at the visible electrode site(s) under both stimulation conditions. The assessor noticed skin redness at the electrode site(s) following stimulation significantly more often following active stimulation than following sham stimulation in both the first session, with a “moderate” level of agreement (κ = 0.512, 95%CI 0.363 to 0.66, p<0.001), and in the second session (κ = 0.677, 95%CI 0.534 to 0.82, p<0.001). Skin redness was noted after 60% of active stimulation sessions and after 1% of sham stimulation sessions.

**Table 2:** Assessors judgements of skin redness at the electrode site, for each stimulation condition.

Participants Judgements	Stimulation condition		Totals
	Active Stimulation	Sham Stimulation	
**Redness noticeable**	59	1	60
**No redness noticable**	39	95	134
**Totals**	98	96	194

### Stimulation Voltage

The median voltage (IQR) at the start of stimulation was 9.2 (7.7 to 11.8). To test whether the initial voltage may have influenced our results the initial stimulation voltage was compared between stimulation conditions (active versus sham) and between participants’ judgements (judged “yes” or “no” to whether they thought they had received active stimulation). No significant difference in voltage was observed for either comparison (Kruskal-Wallace test, by stimulation condition p = 0.693, by participants’ judgement p = 0.377.).

### Missing Data/sensitivity Analysis

To test whether the missing data may have significantly influenced our findings we reanalysed the data on participants’ judgements, substituting all correct responses for the missing values, and then substituting all incorrect responses for the missing values. We did this separately for each stimulation session. The results remained significant, for both the first and second sessions, with both approaches (all incorrect session 1, κ = 0.268, 95% CI 0.088 to 0.456; session 2 κ = 0.699 95%CI 0.559 to 0.839. all correct session 1 κ = 0.29 (95%CI 0.102 to 0.47; session 2 κ = 0.779, 95%CI 0.659 to 0.902).

### Adverse Events

There were no serious adverse events. When the first session was active stimulation, four participants reported an itch that was perceptible throughout the duration of stimulation. One of these participants reported a strong tingling that persisted for the first 2 minutes of stimulation. One participant reported a strong tingling sensation throughout the stimulation and one reported feeling dizzy and drowsy during the stimulation. When the first session was sham, one participant reported mild dizziness during and immediately after, one was unable to tolerate stimulation in the initial 30 second “on” phase due to dizziness and withdrew from the study, although these symptoms had resolved five minutes post stimulation. In the second stimulation session, one participant reported mild dizziness during sham stimulation and one reported feeling drowsy during and immediately after active stimulation.

### Memory Task Data

The memory task was used primarily to distract participants from the true aim of the study. Given that the results have demonstrated that blinding of participants is imperfect it would be problematic to confidently attribute any observed effect on the memory task to the effects of stimulation, or indeed to the placebo effect. As such we did not analyse this data further.

## Discussion

Our results demonstrate that tDCS at 2 mA is not associated with effective blinding when compared with the commonly used sham using this electrode montage and stimulation procedure. For a proportion of tDCS naïve participants, blinding is maintained, but the probability of a participant correctly identifying the stimulation condition is greater than would be expected by chance. Given the high agreement in the second session, the threat to participant blinding appears substantially worse for crossover trials. Participants were more confident in their judgement where they judged that they were receiving active stimulation after the first session though this difference diminished by the second stimulation session.

It is highly likely that the sensory effects of active stimulation were responsible for compromising participant blinding. Familiarity with the experience of stimulation and the ability to compare between sessions amplified this issue after the second stimulation session. Reports of persistent itch or tingling during stimulation in response to the adverse events question are suggestive of this. Most participants probably would not consider these sensations to be adverse effects and so only a minority reported them. Assessor blinding was also compromised in a substantial proportion (60%) of active stimulation in both sessions and this represents an important potential source of bias, regardless of study design, in studies where outcomes are assessed in the immediate post stimulation period.

The current finding has substantial implications for much of the existing literature relating to tDCS. For example, 2 mA intensity and similar electrode montages have been used in almost all trials of tDCS for chronic pain [11], the majority of sham controlled tDCS trials for depression, [6], [13], [14] and all trials of tDCS for reducing cravings [16], [18]–[20]. All of these studies have reported superior efficacy of active stimulation over sham and while some [13], [14] report adequate participant blinding, the issue of assessor blinding was not assessed. While we cannot predict the degree of influence that inadequate blinding may have had in these studies, non-specific effects of interventions are known to be important in such clinical conditions [21], [22]. Further, there is evidence that incomplete blinding leads to exaggerated effects in clinical studies with subjective outcomes [23], and that placebo effects are larger with physical placebo interventions [22]. Thus, we contend that clinical studies that have used 2 mA tDCS should be interpreted with renewed caution. This point is emphasised by the recognised phenomenon that trials of new clinical interventions are often associated with small study effects and a publication bias that influence the evidence base, with a propensity for negative studies to not reach full publication [24], [25].

How might blinding of tDCS at this intensity be improved? Assessor blinding might be ensured by having the participant wear headgear that conceals the area under the electrodes. It is possible that longer ramping times may improve participant blinding but this may not be sufficient where participants are aware of sensations throughout the stimulation period. McFadden et al. [26] demonstrated that the pre-application of topical anaesthetics to electrode sites substantially reduced (but did not abolish) the sensations associated with stimulation, although the same process would be difficult at more posterior locations in participants with hair. Any modified sham protocol will require rigorous testing to ensure adequate blinding.

An alternative approach may be to reduce stimulation intensity. Indeed, it is not clear that higher stimulation intensities are necessary in clinical studies [11]. Effects on cortical excitability have been clearly demonstrated at intensities of 1 mA [1] and there is evidence to suggest that successful participant blinding is achievable under these conditions [9], [10]. Using intensities of 1 mA in future research may represent a more methodologically sound option, although it is plausible that reducing the intensity may reduce potential efficacy. Future studies of tDCS may benefit from other methods to optimise blinding, for example de facto masking [27], in which the treatment is not blinded but both treatments are presented as the active one. De facto masking might be more problematic if a non-stimulation sham is used that carries less credibility with participants but would seem very possible if the “sham” condition is active tDCS over a distinct brain area that is not hypothesized to elicit specific treatment effects.

That we found inadequate blinding using a therapy widely held as blindable [8] raises the possibility that clinical trials of other therapies are vulnerable to similar problems. One obvious example is in trials of TENS, in which the sham condition often involves a deactivated TENS unit and as such there will be distinct differences in the experience of stimulation. It is important to also acknowledge that inadequate blinding is not the only threat to the validity of clinical trials and continued attention should be paid in the design of trials to ensuring rigour in the selection and allocation process of future trials [28].

Our study has some limitations. We did not investigate the perceptual correlates of stimulation in any detail. We took this decision so as to minimise the risk that participants would over-scrutinize the experience of tDCS, which we felt would not accurately reflect the conditions of the average clinical trial. As such we cannot tell with confidence which factors most impacted on blinding. The VAS scale that we used to measure participants’ confidence in their judgements has not been specifically validated for that task and may have lacked sensitivity and validity, although this would not confound our results so much as reduce our power to detect non-blinding. The predominance of female participants might plausibly have affected our results. There is some evidence that differences exist between males and females, in pain threshold and pain evoked by a standard noxious stimulus, but the nature of the difference depends upon the type of stimulus and the context in which it is tested (see [29]for a review). There is also some debate as to whether pain thresholds vary in females according to stage of their menstrual cycle [30]–[32]. However, randomisation of the order of stimulation should mitigate any potential impact of these issues on our data. Finally the persistence of noticeable skin redness that persists beyond the immediate post-stimulation period represents a further risk to participant blinding and suggests that our results may underestimate the scale of the problem.

In conclusion, contrary to the assumption of blinding, which underpins the growing support of tDCS for clinical conditions, our data show that both participant and assessor blinding is compromised at 2 mA intensity when using this electrode montage and stimulation procedure. The findings have important implications for the interpretation of studies which have utilised this approach and for the design of future tDCS studies.
